# Supplementary material for: NKG2D upregulation sensitizes tumors to combined anti-PD1 and anti-VEGF therapy and prevents hearing loss
Source: Nat Commun. 2026 Feb 11;17:1148. doi: 10.1038/s41467-026-68865-8 (PMC12894996; doi:10.1038/s41467-026-68865-8)
Supplement: Supplementary file 1 — Supplementary Information [file 41467_2026_68865_MOESM1_ESM.pdf]

NKG2D Upregulation Sensitizes Tumors to Combined Anti-PD1 and Anti-VEGF Therapy and Prevents Hearing Loss

Authors

Simeng Lu[1,a], Zhenzhen Yin[1,a], Limeng Wu[1], Yao Sun[1,2], Jie Chen[1], Lai Man Natalie Wu[3], Janet L. Oblinger[4], Day Caven Blake[1], Bingyu Xiu[1], Lukas D. Landegger[5], Richard Seist[5], William Ho[1], Adam P. Jones[1], Alona Muzikansky[6], Konstantina Stankovic[5], Scott R. Plotkin[7], Long-Sheng Chang[4], and Lei Xu[1,*]

Affiliations

[1] Edwin L. Steele Laboratories, Department of Radiation Oncology, Massachusetts General Hospital, Harvard Medical School, Boston, MA, 02114, USA

[2] Medpace, Inc., Cincinnati, OH, 45227, USA

[3] Center for Childhood Cancer, Abigail Wexner Research Institute at Nationwide Children's Hospital and Department of Pediatrics, The Ohio State University, Columbus, OH, 43215, USA

[4] Department of Otolaryngology – Head and Neck Surgery and Department of Neurosurgery, Stanford University School of Medicine, Stanford, CA, 94305, USA

[5] Biostatistics Center, Massachusetts General Hospital, Harvard Medical School, Boston, MA, 02114, USA

[6] Department of Neurology and Cancer Center, Massachusetts General Hospital, Harvard Medical School, Boston, MA, 02114, USA

Supplementary Figures

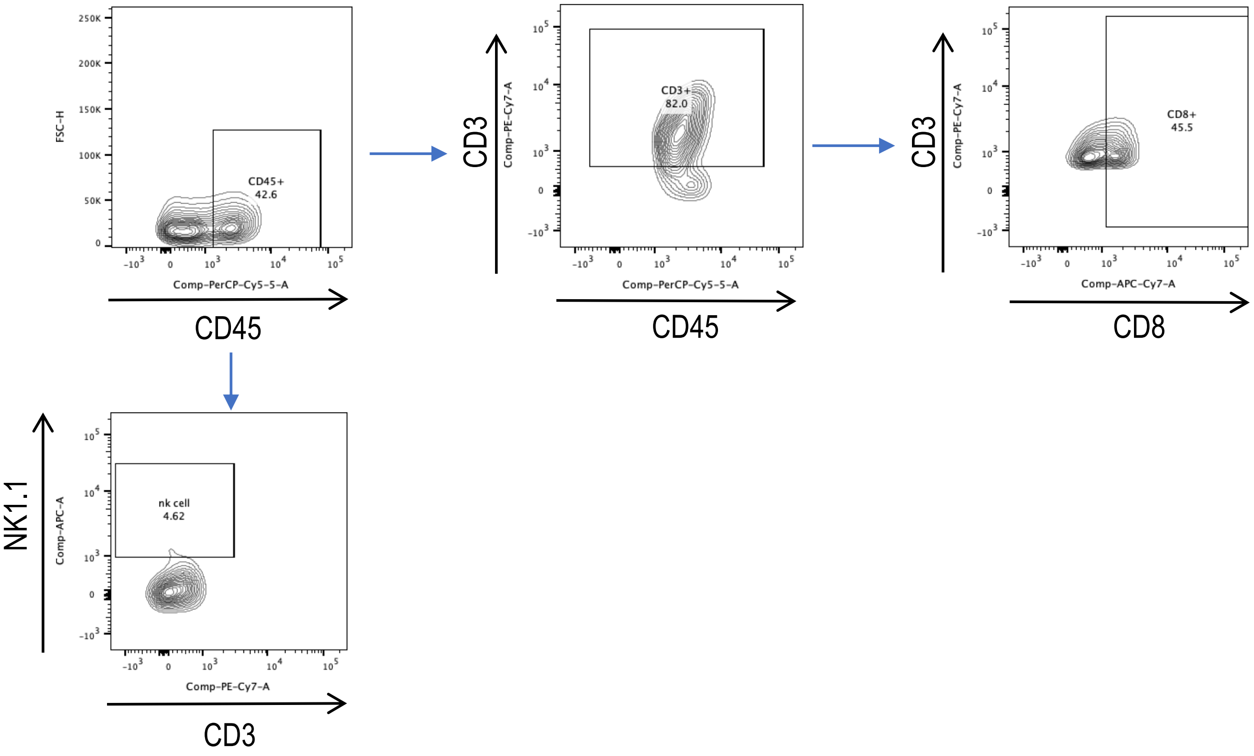

Figure S1. αVEGF treatment increases intratumoral infiltration of immune effector cells in the mouse schwannoma model. Representative gating strategy for CD8$^+$ T cells and NK cells of flow cytometry analysis. Live single cells were first gated for CD45$^+$ leukocytes. Within the CD45$^+$ population, CD3$^+$ T cells were identified, followed by further discrimination of CD8$^+$ T cells from total CD3$^+$ cells. Separately, NK cells (CD3$^-$NK1.1$^+$) were gated from the CD45$^+$CD3$^-$ population. The percentage of positive cells within each gate is indicated on the plots.

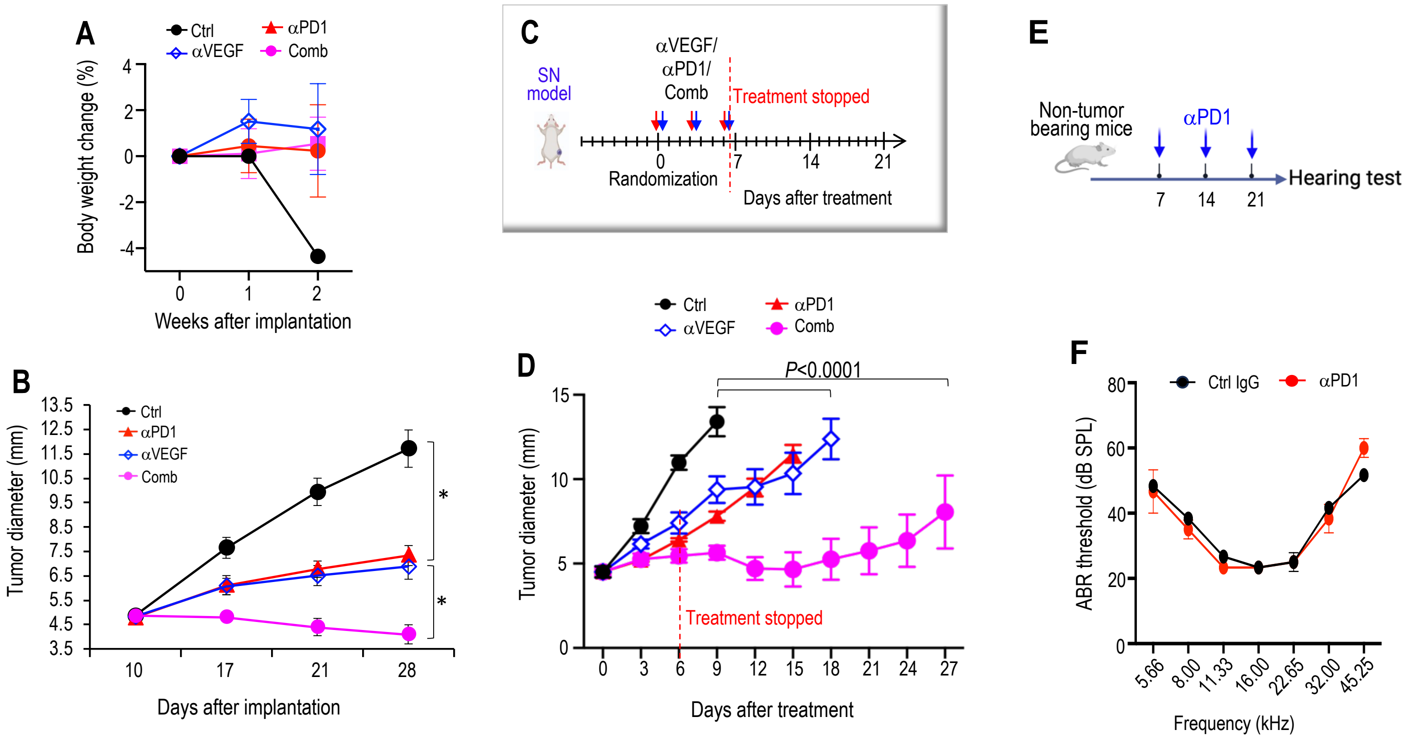

Figure S2. Combined αVEGF treatment enhances αPD1 efficacy in the mouse schwannoma models.

(A) Bodyweight of mice was measured at the indicated timepoints and expressed as a percentage of the initial body weight before tumor implantation on day 0. No significant bodyweight loss (defined as >15% change) was observed in any treatment group.

(B) In the sciatic nerve SC4 model, tumor-bearing mice were treated with control IgG, αPD1 (200 mg/mice), αVEGF (2.5 mg/kg), or combined αPD1 and αVEGF. Tumor diameter was measured by caliper every 3 days post-treatment. *P<0.0001.

(C) Diagram showing the timeline of αVEGF and αPD1 combination treatment in mice bearing *Nf2*$^{-/-}$ tumors in the sciatic nerve. We discontinued all treatments after administering three doses. The schematic in panel C was created in BioRender Xu, L. (2026) http://BioRender.com/yltfaz6.

(D) Tumor diameter was measured by caliper every 3 days post-treatment.

(E) Diagram showing the timeline of αPD1 treatment in non-tumor bearing mice. Hearing test was performed 21 days post treatment. The schematic in panel E was created in BioRender Xu, L. (2026) http://BioRender.com/ffepnmy.

(F) Hearing function test was carried out by testing the ABR threshold as a function of frequency in mice treated with control IgG (Ctrl) or αPD1.

All animal studies are representative of at least three independent experiments with graphs depicting the mean±SEM (N=18 mice/group). Differences in sciatic nerve tumor growth was analyzed using repeated-measures two-way ANOVA. ABR thresholds were analyzed with a linear mixed-effects model.

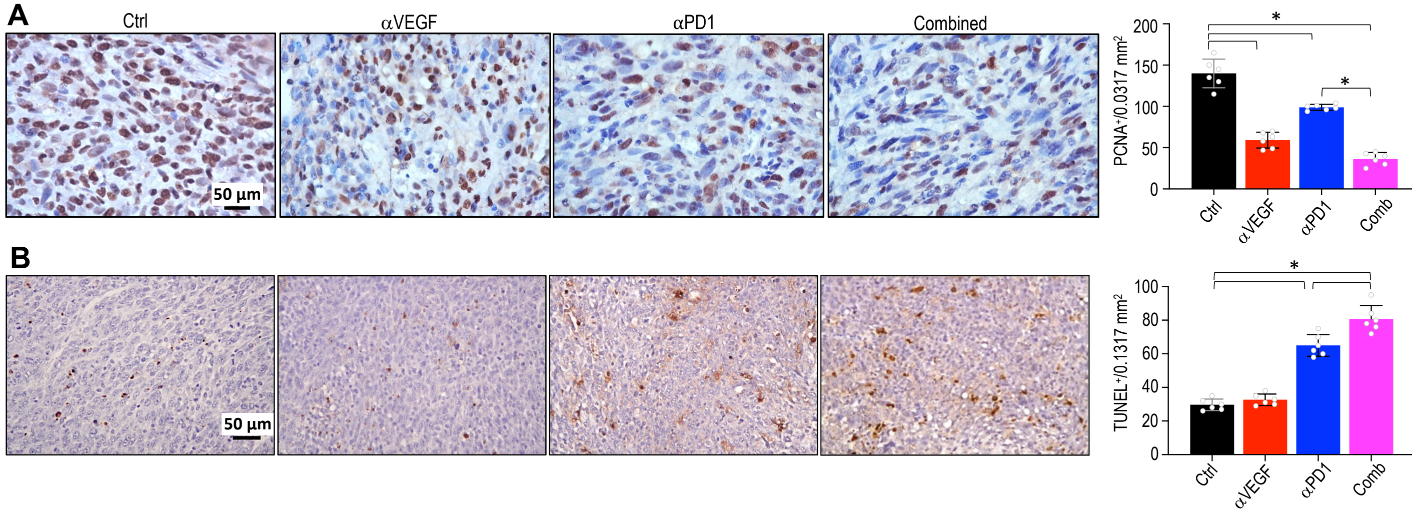

Figure S3. Combined αVEGF and αPD1 treatment reduces proliferating and increases apoptotic tumor cells compared to control and monotherapies in *Nf2*$^{-/-}$ model.

(A) Representative images of PCNA staining for proliferating tumor cells. The number of PCNA^{+} cells were manually counted.

(B) Representative images of TUNEL staining for apoptotic cells. The number of TUNEL^{+} cells were manually counted.

Image quantification are presented as mean ± SD, and analyzed using two-way Student's t-test and the Mann-Whitney test.

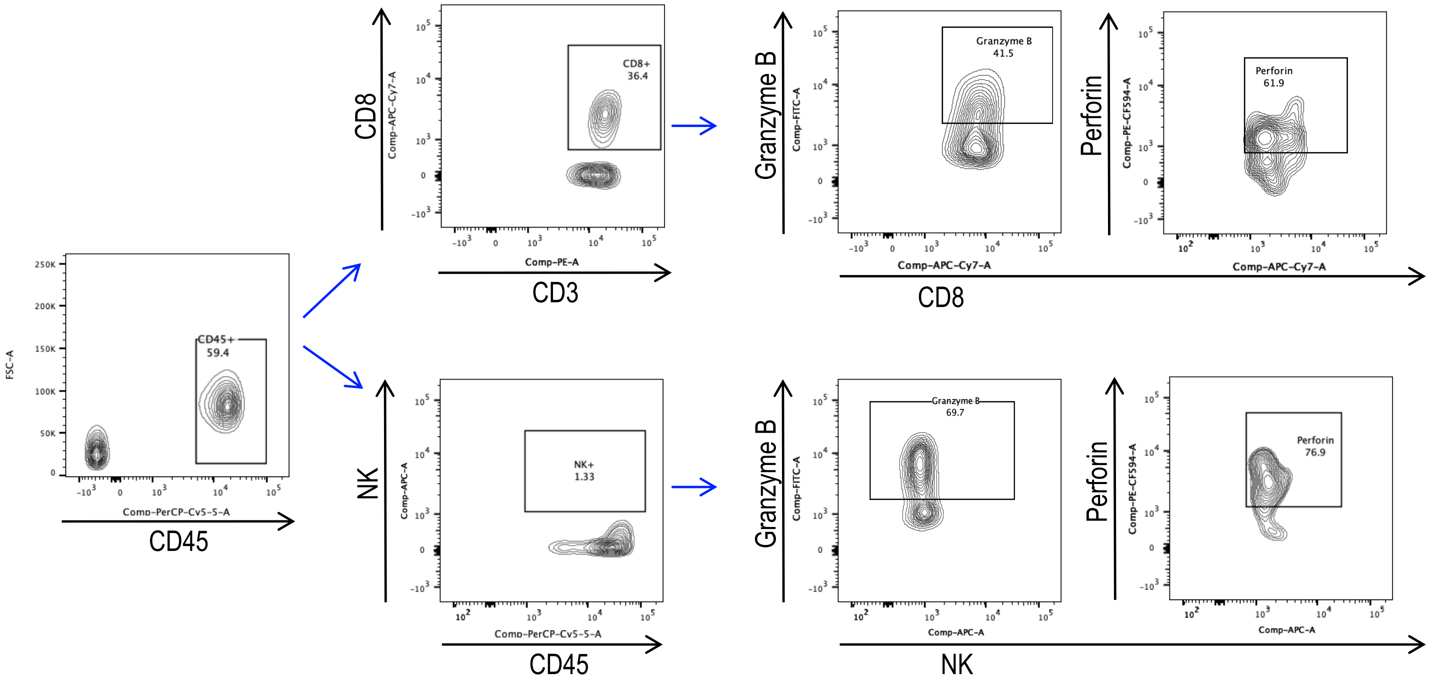

Figure S4. αVEGF treatment increases the proportion of CD8⁺ T cells and NK cells expressing granzyme B and perforin. Representative flow cytometry plots showing the sequential gating strategy used to quantify the proportion of $CD8^+$ T cells and NK cells expressing granzyme B and perforin. Live single cells were first gated on $CD45^+$ leukocytes. Within the $CD45^+$ population, $CD8^+$ T cells (top row) and NK cells (bottom row) were subsequently analyzed for intracellular expression of Granzyme B and Perforin as markers of cytotoxic effector function. The percentage of positive cells within each gate is indicated on the plots.

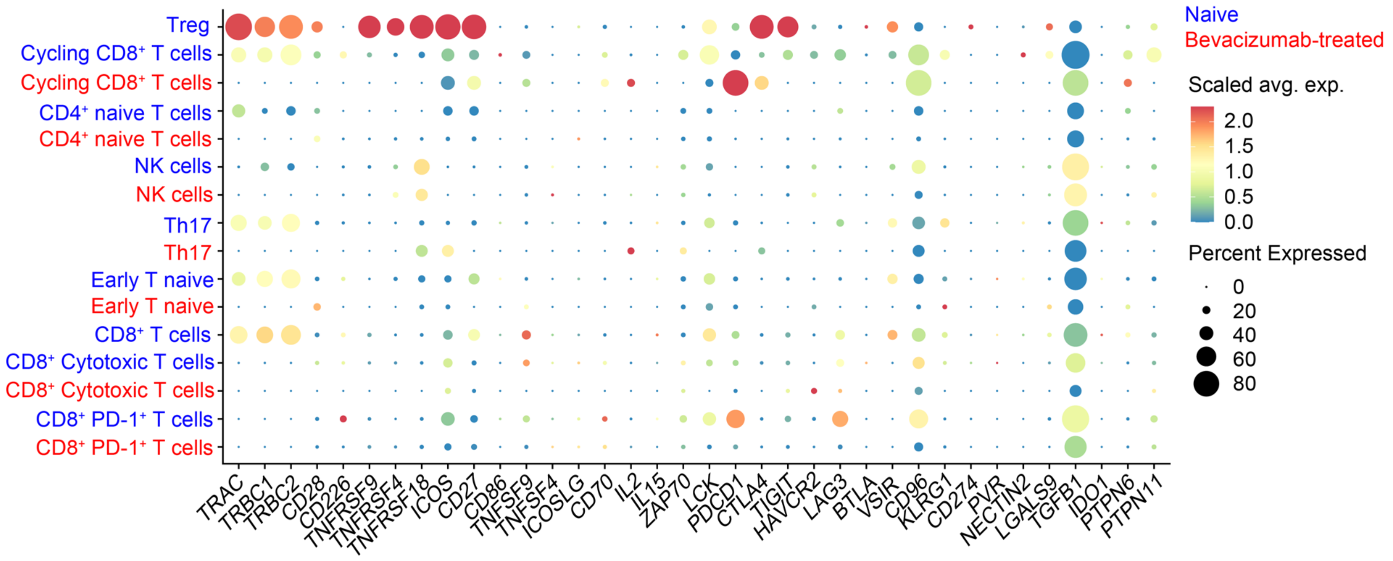

Figure S5. T cell activation, co-stimulatory, and co-inhibitory receptor landscape in Naïve vs. bevacizumab-treated *NF2*-SWN patient samples. Dot plot showing scaled average expression (color) and percent of cells expressing each gene (dot size) across major T cell and NK cell subsets. Rows represent individual immune cell populations in naïve samples (blue label) and bevacizumab-treated samples (red labels).

Table S1. Antibody panels.

Treatment antibodies

Target	Clone	Company	Catalog Number
Anti-VEGF	B20-4.4	Genentech	Under MTA
Isotype control IgG	2A3	BioXCell	BE0089
Anti-PD1	RMP1-14	BioXCell	BE0146
Anti-CD8	2.43	BioXCell	BE0061
Anti-NK	PK136	BioXCell	BE0036
Anti-NKG2D	CX5	BioXCell	BE0034

Flow cytometry antibodies

Surface Markers	Clone	Conjugate	Catalog Number	Dilution
CD11b	M1/70	APC-Cy7	BioLegend 101212	1:200
CD3	17A2	PE-cy7	BioLegend 100219	1:200
CD4	GK1.5	FITC	BioLegend 100405	1:200
CD45	30-F11	PerCP	BioLegend 103130	1:200
CD8	53-6.7	APC-cy7	BioLegend 100713	1:200
Gr1	RB6-8C5	PE	BioLegend 108408	1:200
NK1.1	S17016D	APC	BioLegend 156505	1:200
Granzyme B	GB11	FITC	ThermoFisher 12-8898-82	1:100
Perforin	eBioOMAK-D	PE/Dazzl 594	ThermoFisher 14-9392-82	1:100

Staining antibodies

Markers	Clone	Catalog Number	Dilution
αSMA	1A4	Sigma-Aldrich 202M-97	1:200
CD31	2H8	Millipore MAB1398Z	1:200
PCNA	PC10	Abcam 13-3900	1:1000
