# Supplementary material for: NKG2D upregulation sensitizes tumors to combined anti-PD1 and anti-VEGF therapy and prevents hearing loss
Source: Nat Commun. 2026 Feb 11;17:1148. doi: 10.1038/s41467-026-68865-8 (PMC12894996; doi:10.1038/s41467-026-68865-8)
Supplement: Supplementary file 2 — Reporting Summary [file 41467_2026_68865_MOESM2_ESM.pdf]

nature portfolio

Corresponding author(s): Lei Xu

Last updated by author(s): Dec 31, 2025

Reporting Summary

Nature Portfolio wishes to improve the reproducibility of the work that we publish. This form provides structure for consistency and transparency in reporting. For further information on Nature Portfolio policies, see our Editorial Policies and the Editorial Policy Checklist.

Statistics

For all statistical analyses, confirm that the following items are present in the figure legend, table legend, main text, or Methods section.

n/a	Confirmed	
☐	☒	The exact sample size (n) for each experimental group/condition, given as a discrete number and unit of measurement
☐	☒	A statement on whether measurements were taken from distinct samples or whether the same sample was measured repeatedly
☐	☒	The statistical test(s) used AND whether they are one- or two-sided<br>*Only common tests should be described solely by name; describe more complex techniques in the Methods section.*
☐	☒	A description of all covariates tested
☒	☐	A description of any assumptions or corrections, such as tests of normality and adjustment for multiple comparisons
☐	☒	A full description of the statistical parameters including central tendency (e.g. means) or other basic estimates (e.g. regression coefficient) AND variation (e.g. standard deviation) or associated estimates of uncertainty (e.g. confidence intervals)
☐	☒	For null hypothesis testing, the test statistic (e.g. F, t, r) with confidence intervals, effect sizes, degrees of freedom and P value noted<br>*Give P values as exact values whenever suitable.*
☒	☐	For Bayesian analysis, information on the choice of priors and Markov chain Monte Carlo settings
☒	☐	For hierarchical and complex designs, identification of the appropriate level for tests and full reporting of outcomes
☒	☐	Estimates of effect sizes (e.g. Cohen's d, Pearson's r), indicating how they were calculated

Our web collection on statistics for biologists contains articles on many of the points above.

Software and code

Policy information about availability of computer code

Data collection	Confocal image acquisition was performed on an Olympus IX81 confocal microscope with Fluoviewer FV10-ASW4.2 software.ABR threshold data acquisition was achieved using custom LabVIEW software operating on a PXI chassis from National Instruments Corp.
Data analysis	RNASeq data analysis: For computational analysis, we used Cellranger v5.0.1 to align reads to the hg19 human reference sequence. For each sample dataset, unsupervised clustering was performed using the R package Seurat (version 4) (https://www.satijalab.org/seurat; https://www.github.com/satijalab/seurat). Histological evaluation using digital quantitative image analysis was performed using ImageJ. All statistical analyses were carried out using GraphPad Prism Software version 9.

For manuscripts utilizing custom algorithms or software that are central to the research but not yet described in published literature, software must be made available to editors and reviewers. We strongly encourage code deposition in a community repository (e.g. GitHub). See the Nature Portfolio guidelines for submitting code & software for further information.

Data

Policy information about availability of data

All manuscripts must include a data availability statement. This statement should provide the following information, where applicable:

- Accession codes, unique identifiers, or web links for publicly available datasets
- A description of any restrictions on data availability
- For clinical datasets or third party data, please ensure that the statement adheres to our policy

All data and the supplementary materials from this study are included in this manuscript and are available after publication upon request from the corresponding author. Sequencing data will be deposited in GEO and will be available upon publication.

Research involving human participants, their data, or biological material

Policy information about studies with human participants or human data. See also policy information about sex, gender (identity/presentation), and sexual orientation and race, ethnicity and racism.

Reporting on sex and gender	N/A
Reporting on race, ethnicity, or other socially relevant groupings	N/A
Population characteristics	N/A
Recruitment	N/A
Ethics oversight	N/A

Note that full information on the approval of the study protocol must also be provided in the manuscript.

Field-specific reporting

Please select the one below that is the best fit for your research. If you are not sure, read the appropriate sections before making your selection.

☒ Life sciences ☐ Behavioural & social sciences ☐ Ecological, evolutionary & environmental sciences

For a reference copy of the document with all sections, see nature.com/documents/nr-reporting-summary-flat.pdf

Life sciences study design

All studies must disclose on these points even when the disclosure is negative.

Sample size	Sample sizes are included in each figure legend
Data exclusions	no data were excluded from the analyses
Replication	Replication information is included in each figure legend
Randomization	Randomization information is included in each figure legend
Blinding	Investigator was blinded during data collection.

Reporting for specific materials, systems and methods

We require information from authors about some types of materials, experimental systems and methods used in many studies. Here, indicate whether each material, system or method listed is relevant to your study. If you are not sure if a list item applies to your research, read the appropriate section before selecting a response.

Materials & experimental systems

n/a	Involved in the study
☐	☒ Antibodies
☐	☒ Eukaryotic cell lines
☒	☐ Palaeontology and archaeology
☐	☒ Animals and other organisms
☒	☐ Clinical data
☒	☐ Dual use research of concern
☒	☐ Plants

Methods

n/a	Involved in the study
☒	☐ ChIP-seq
☐	☒ Flow cytometry
☒	☐ MRI-based neuroimaging

Antibodies

Antibodies used

Perforin Cell Signaling Technology N/A (rabbit polyclonal) WB / Flow 1:500
Granzyme B Cell Signaling Technology N/A (rabbit polyclonal) WB / Flow 1:500
β-Actin Sigma-Aldrich AC-15 WB 1:5000
PCNA (Proliferating Cell Nuclear Antigen) Abcam PC10 IHC 1:1000
CD31 (PECAM-1) Millipore 2H8 IHC / IF 1:200
α-Smooth Muscle Actin-Cy3™ Sigma-Aldrich 1A4 IF 1:200
TUNEL (ApopTag® Kit) Millipore N/A (kit) IHC (Apoptosis) Per protocol
FITC-Lectin (Tomato lectin) Vector Laboratories N/A (lectin) Perfusion labeling 2 mg/kg (i.v.)
αPD-1 (anti-mouse PD-1) BioXCell RMP1-14 In vivo / IF (FITC-labeled) 200 μg/mouse i.p.
Isotype IgG control BioXCell 2A3 In vivo control 200 μg/mouse i.p.
αVEGF (B20-4.1.1) Genentech B20-4.1.1 In vivo 2.5 mg/kg i.p. weekly
CD45 BD Biosciences 30-F11 Flow cytometry 1:200
CD4 BD Biosciences RM4-5 Flow cytometry 1:200
CD8a BD Biosciences 53-6.7 Flow cytometry 1:200
NK1.1 BD Biosciences PK136 Flow cytometry 1:200
Gr-1 (Ly6G/Ly6C) BD Biosciences RB6-8C5 Flow cytometry 1:200
CD11b BD Biosciences M1/70 Flow cytometry 1:200
Granzyme B (Flow) BD Biosciences GB11 Flow cytometry 1:100
Perforin (Flow) BD Biosciences S16009A Flow cytometry 1:100

Validation

Antibody validation provided by manufacture.

Eukaryotic cell lines

Policy information about cell lines and Sex and Gender in Research

Cell line source(s)

Mouse Nf2-/- Schwann cells and mouse SC4 Schwannoma cells were gifts from Dr. Vijaya Ramesh, Massachusetts General Hospital, MGH .

Authentication

N/A

Mycoplasma contamination

We confirm cells were tested for mycoplasma contamination periodically, and are free from mycoplasma contamination.

Commonly misidentified lines (See ICLAC register)

No misidentified cells used.

Animals and other research organisms

Policy information about studies involving animals; ARRIVE guidelines recommended for reporting animal research, and Sex and Gender in Research

Laboratory animals

C57/FVB mice

Wild animals

N/A

Reporting on sex

Both male and female mice (1:1 ratio) aged 8-12 weeks old were used to ensure sufficient statistical power and to examine any potential sex-related differences.

Field-collected samples

N/A

Ethics oversight

Animal experiments were conducted in accordance with the protocol approved by the Institutional Animal Care and Use Committee of MGH.

Note that full information on the approval of the study protocol must also be provided in the manuscript.

Plants

Seed stocks | N/A

Novel plant genotypes | N/A

Authentication | N/A

Flow Cytometry

Plots

Confirm that:

☒ The axis labels state the marker and fluorochrome used (e.g. CD4-FITC).

☒ The axis scales are clearly visible. Include numbers along axes only for bottom left plot of group (a 'group' is an analysis of identical markers).

☒ All plots are contour plots with outliers or pseudocolor plots.

☒ A numerical value for number of cells or percentage (with statistics) is provided.

Methodology

Sample preparation | Sample preparation information are included in Methods

Instrument | Instrument information are included in Methods

Software | Software information are included in Methods

Cell population abundance | N/A

Gating strategy | Gating strategy are included in Supplemental figures.

☒ Tick this box to confirm that a figure exemplifying the gating strategy is provided in the Supplementary Information.
